# Editorial: Advances in brain imaging and stimulation methods for cognitive function investigation, volume II

**DOI:** 10.3389/fnhum.2025.1611543

**Published:** 2025-05-01

**Authors:** Mengze Xu, Yuwen He, Yujing Huang, Zhen Yuan

**Affiliations:** ^1^Department of Biology, Faculty of Arts and Sciences, Beijing Normal University, Zhuhai, China; ^2^Centre for Cognitive and Brain Sciences, University of Macau, Zhuhai, Macau SAR, China; ^3^Faculty of Health Sciences, University of Macau, Zhuhai, Macau SAR, China

**Keywords:** brain imaging, brain stimulation, cognitive function, neuromodulation, multimodal integration

## Technological innovation: from high-resolution imaging to targeted neuromodulation

**Figure 1:**
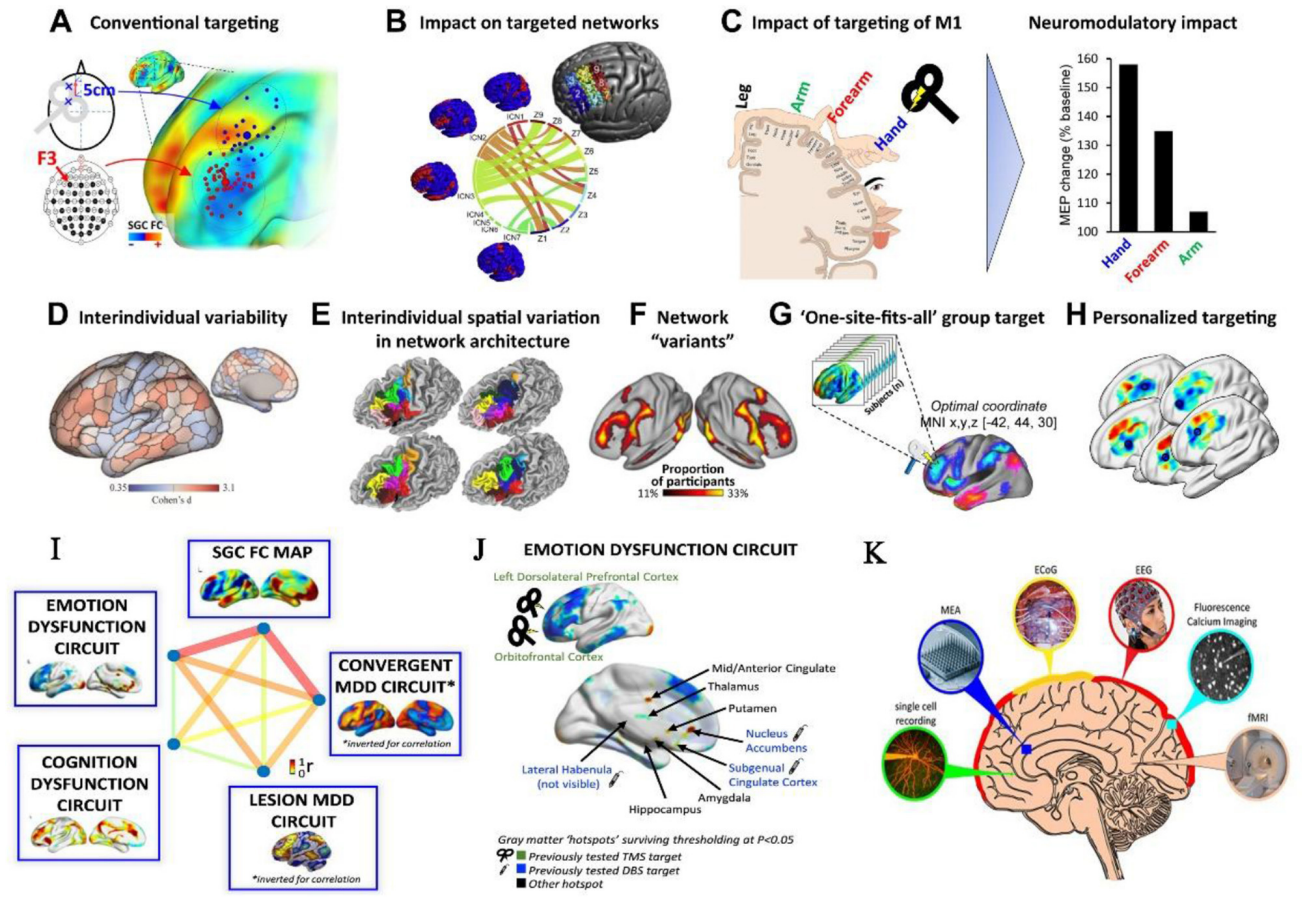
Representative brain imaging and stimulation methods for personalized interventions targeting cognitive and affective functions. **(A–H)** Rationale for personalized targeting based on brain connectivity. **(I, J)** Cognitive and affective circuits that can be targeted by intervention. Copyright © 2024 Elsevier B.V. **(K)** Commonly used techniques for recording brain activity. Copyright © The University of Queensland.

The quest for high spatiotemporal resolution in brain imaging has driven remarkable advances. For instance, multimodal approaches integrating electroencephalography (EEG) with functional magnetic resonance imaging (fMRI) or near-infrared spectroscopy (NIRS) now enable simultaneous mapping of hemodynamic and electrophysiological correlates of cognition (Gao et al., 2022). Meanwhile, innovations in network neuroscience, such as whole-brain connectivity analyses (Figure 1), have unlocked personalized targeting strategies for non-invasive neuromodulation (Cash and Zalesky, 2024).

In the realm of neuromodulation, non-invasive techniques like transcranial magnetic stimulation (TMS) and transcranial direct current stimulation (tDCS) continue to dominate due to their safety and reversibility. A systematic review in this Research Topic, *Effects of transcranial direct current stimulation on modulating executive functions in healthy populations*, highlights tDCS-mediated enhancements in executive functions (e.g., working memory and inhibitory control) through prefrontal cortex modulation (You et al.). However, effect variability tied to stimulation parameters and interindividual differences underscores the need for precision protocols. Similarly, *a pooled analysis of transcutaneous auricular vagus nerve stimulation (taVNS)* side effects of non-invasive taVNS reaffirms its safety profile while advocating for rigorous dose optimization and longitudinal monitoring (Giraudier et al.).

## Clinical translation: bridging mechanisms and interventions

The synergy between imaging and stimulation holds transformative potential for cognitive disorders (Figure 1). A pivotal study in this Research Topic, *Effect of continuous theta burst stimulation on the glymphatic system and cognition in cerebral small vessel disease*, demonstrates how continuous theta burst stimulation (cTBS) modulates glymphatic clearance and default-mode network connectivity, improving information processing speed and executive function in patients (Dai et al.). Such findings and neuroimaging biomarkers (e.g., functional connectivity strength) offer objective metrics for evaluating therapeutic efficacy.

However, clinical translation demands careful navigation of neuroplasticity and ethical risks. A study in this Research Topic, *The safety and efficacy of applying a high-current temporal interference electrical stimulation in humans*, addressed this Research Topic, evaluating the safety and efficiency of a proposed high-current temporal interference electrical stimulation (Wang et al.). Personalized neuromodulation protocols must balance innovation with ethical responsibility to mitigate unintended consequences. Moreover, the application of imaging biomarkers for early diagnosis (e.g., functional connectivity anomalies in Alzheimer's disease) requires standardized, reproducible methodologies across multicenter datasets.

## Challenges and future directions

Despite progress being achieved in this field, there are some critical gaps that warrant researchers' attention.

1. Multimodal data integration. How can we unify structural, functional, and metabolic imaging data to build cross-scale models of cognition? Machine learning and radiomics approaches may offer solutions (Hua et al., 2025). The coupling and interaction among different modalities of data might also carry interesting process information (Zeng et al., 2024).

2. Dynamic brain imaging in naturalistic contexts and wearable intervention devices. Capturing neural dynamics during real-world cognitive tasks remains technically challenging, necessitating advances in portable devices and real-time analytics. Meanwhile, wearable intervention devices can enable broader utility (Qi et al., 2025).

3. Validating causal mechanisms. Combining neuromodulation with pre-post imaging (e.g., TMS-EEG or tDCS-fMRI) can strengthen causal inferences (Bergmann and Hartwigsen, 2021). For example, studies pairing TMS with event-related potentials (ERPs) in this Research Topic reveal neuroplasticity markers in traumatic brain injury recovery.

## Conclusion

The four studies in this Research Topic exemplify the diversity and utility of modern brain imaging and stimulation tools, spanning methodological refinement, clinical validation, and safety assessment. Moving forward, research must prioritize standardization (aligned with FAIR principles), interdisciplinary collaboration (e.g., engineering and cognitive science), and ethical frameworks to ensure responsible innovation. By addressing these challenges, we move closer to the ultimate goal of decoding cognition to enable precision interventions, paving the way for individualized therapies in neuropsychiatric disorders.
